# Intervolume analysis to achieve four-dimensional optical microangiography for observation of dynamic blood flow

**DOI:** 10.1117/1.JBO.21.3.036005

**Published:** 2016-03-11

**Authors:** Wei Wei, Jingjiang Xu, Utku Baran, Shaozhen Song, Wan Qin, Xiaoli Qi, Ruikang K. Wang

**Affiliations:** a University of Washington, Department of Bioengineering, 3720 15th Avenue NE, Seattle, Washington 98195, United States; b University of Washington, Department of Electrical Engineering, 185 Stevens Way, Seattle, Washington 98195, United States

**Keywords:** optical coherence tomography angiography, optical microangiography, intervolume, four-dimensional, dynamic blood flow, Fourier domain mode locking, microelectromechanical system

## Abstract

We demonstrate *in vivo* volumetric optical microangiography at ∼200  volumes/s by the use of 1.6 MHz Fourier domain mode-locking swept source optical coherence tomography and an effective 36 kHz microelectromechanical system (MEMS) scanner. We propose an intervolume analysis strategy to contrast the dynamic blood flow signal from the static tissue background. The proposed system is demonstrated by imaging cerebral blood flow in mice *in vivo*. For the first time, imaging speed, sensitivity, and temporal resolution become possible for a direct four-dimensional observation of microcirculations within live body parts.

Optical coherence tomography (OCT)^1^ is a promising noninvasive and high resolution imaging modality for three-dimensional (3-D) imaging of biological tissues.^2^ Its functional extension of OCT-based angiography has been becoming increasingly clinically important due to its ability to provide volumetric microvascular networks *in vivo* without a need of exogenous contrast dyes.^3^ Based on inter-A-line analysis or inter-B-frame analysis, numerous OCT angiography (OCTA) algorithms have been proposed to contrast functional microvasculature, such as optical microangiography (OMAG),^4^ speckle variance,^5^ phase variance,^6^ and correlation mapping.^7^ Using ultrahigh sensitivity optical microangiography (UHS-OMAG), an unprecedented sensitivity to 4  μm/s was achieved.^8^ While promising, these popular angiographic techniques are currently limited to 3-D imaging due to the constraints of available light sources/detectors that define the system A-scan rate, and scanners that dictate how fast the probe beam can be scanned to achieve 3-D imaging. On the other hand, the sensitivity to blood flow measurement is directly related to the time interval Δt for analysis. The shorter Δt translates to a lower sensitivity of flow measurement. In practice, one often selects to use Δt in a range of ∼2 to 5 ms for angiographic data analysis to contrast slow blood flows, for example, in capillary vessels, within tissue *in vivo*.^3^

With the development of new swept laser sources, OCT imaging speed has moved into a new era of multi-MHz imaging rate. Blatter et al.^9^ acquired human retinal microvasculature by using a Fourier domain mode locking (FDML) swept source OCT (SS-OCT) system operating at an A-line rate of 1.68 MHz and a B-frame rate of 560 fps (Δt=∼1.8  ms), which took ∼7  s to finish one 3-D scan. With the increase of the B-frame rate up to 3.4 kHz (Δt=∼0.3  ms), Zhi et al.^10^ demonstrated four-dimensional (4-D) microangiography of mouse ear microcirculatory tissue bed at 4.7  volumes/s with 1.62 MHz A-line rate. However, these newest developments are still limited to the interframe analysis for blood flow imaging. While the B-frame rate is increased (and therefore, the time interval Δt is decreased), the sensitivity to blood flow measurement is inevitably reduced. Although one may intentionally slow down the scanner to acquire B-scans in order to increase sensitivity, the drawback is the dramatic increased total data acquisition time for each 3-D volume, leading to a temporal resolution that would make it difficult to observe the dynamic behavior of blood flow in 3-D. We noticed that a 4-D speckle variance technique was recently reported to contrast the blood vessels within the yolk sac of a mouse embryo.^11^ However, the demonstrated volumetric imaging speed was ∼43 (Δt=23  ms), which is too slow to observe the dynamic behavior of the blood flow.

Here, we propose a volumetric optical microangiography (vOMAG) method by using intervolume analysis as a useful extension to the current OCT-based angiography techniques. The vOMAG algorithm to contrast the motion signal from tissue background is applied along continuous volumes, rather than along B-frames (slow scanning direction) as in the conventional approach. To achieve the intervolume analysis, we develop a high-speed OCT system by combining an FDML laser source sweeping at ∼1.62  MHz with a microelectromechanical system (MEMS) scanner working at an effective frame rate of 36 kHz. This system setup enables a volumetric imaging rate at ∼200  volumes/s, leading to a 3-D temporal resolution of ∼5  ms that meets the requirement of contrasting slow blood flows in capillary vessels. To the best of our knowledge, this is the first demonstration that utilizes the intervolume analysis to perform high-speed and high-definition angiography of microcirculatory tissue beds *in vivo*.

The schematic setup of the high-speed OCT system is shown in Fig. 1. An FDML swept laser (FDML-1310-4B-APC, Optores GmbH) was used as the OCT light source, which was tuned at a central wavelength of 1308 nm with a 3 dB spectral scanning range of 110 nm, giving an A-line scanning rate of 1.6217 MHz. Output power of 30 mW from the FDML source was fiber-coupled into an OCT system engine. In the engine, the light was first split by a 90:10 fiber coupler with 10% power going to the reference arm. The remaining 90% power was further divided by a 99:1 fiber coupler into a sample arm and a recalibration arm, respectively. The reference arm and the sample arm formed a master interferometer to provide the spectral interference signal to enable the imaging of sample. The recalibration arm together with the reference arm formed a slave interferometer that was used to resample the interference signal from the master interferometer so that the spectral interference signal emerging from the sample is represented in linear k-space.^12^ In the sample arm, a 5× OCT scan lens (LSM03, Thorlabs Inc.) focused the light beam into the sample with a theoretical beam size of ∼29  μm at the focal point. To achieve a high B-frame rate, a MEMS scanner (PicoP, MicroVision Inc.)^13^ was used to scan the beam spot. The MEMS scanner was driven by an 18 kHz sinusoid wave generated from an arbitrary waveform generator (AWG520, Tektronix) synchronized with the light source. Considering the bidirectional movement of the scanner, the effective frame rate was therefore 36,000  frames/s (fps). The configuration of the scanning system in the sample arm was so designed that it provided a B-scan size of 1 mm along the fast scanning direction, that is, the x-direction (consisting of ∼45 A-scans). A galvanometer scanner (6215H, Cambridge Technology) was used to provide the slow axis, that is, y scanning, driven by a continuous ramp waveform at 200 Hz. There were 180 B-frames (considering the bidirectional fast scan), covering 2 mm, in the slow axis. This scanning protocol [as shown in Fig. 2(a)] provided a sampling interval of ∼22  μm in both the x- and y-directions, which is enough to resolve small vessels at ∼30  μm. Reflected light from the reference arm and the sample arm interfered at a 50/50 coupler and was then detected by a 1.6 GHz dual balanced photodetector (PDB480C-AC, Thorlabs Inc.). The inference signal was sampled and digitized at 1.5  GS/s by a 12 bits A/D acquisition card (ATS9360, Alazartech). The incident light power on the sample was measured at ∼13  mW, providing a measured system sensitivity of ∼101  dB at the focus depth position. The acquired signal (raw data) was further processed through third-order Hermite kernel resampling, fast Fourier transform, and logarithmic compression,^12^ and then piled into final 3-D structure datasets.

**Fig. 1:**
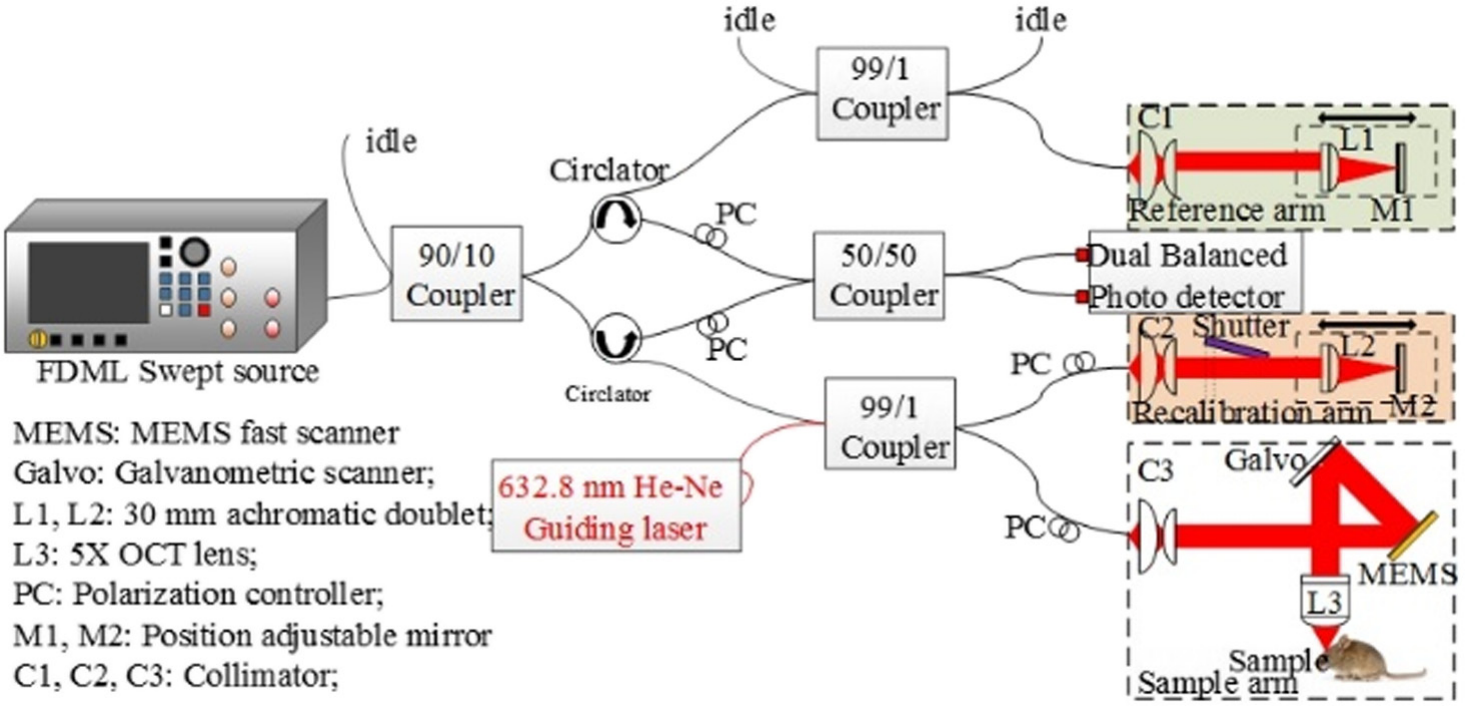
Schematic of the OCT system with FDML SS and MEMS scanner.

**Fig. 2:**
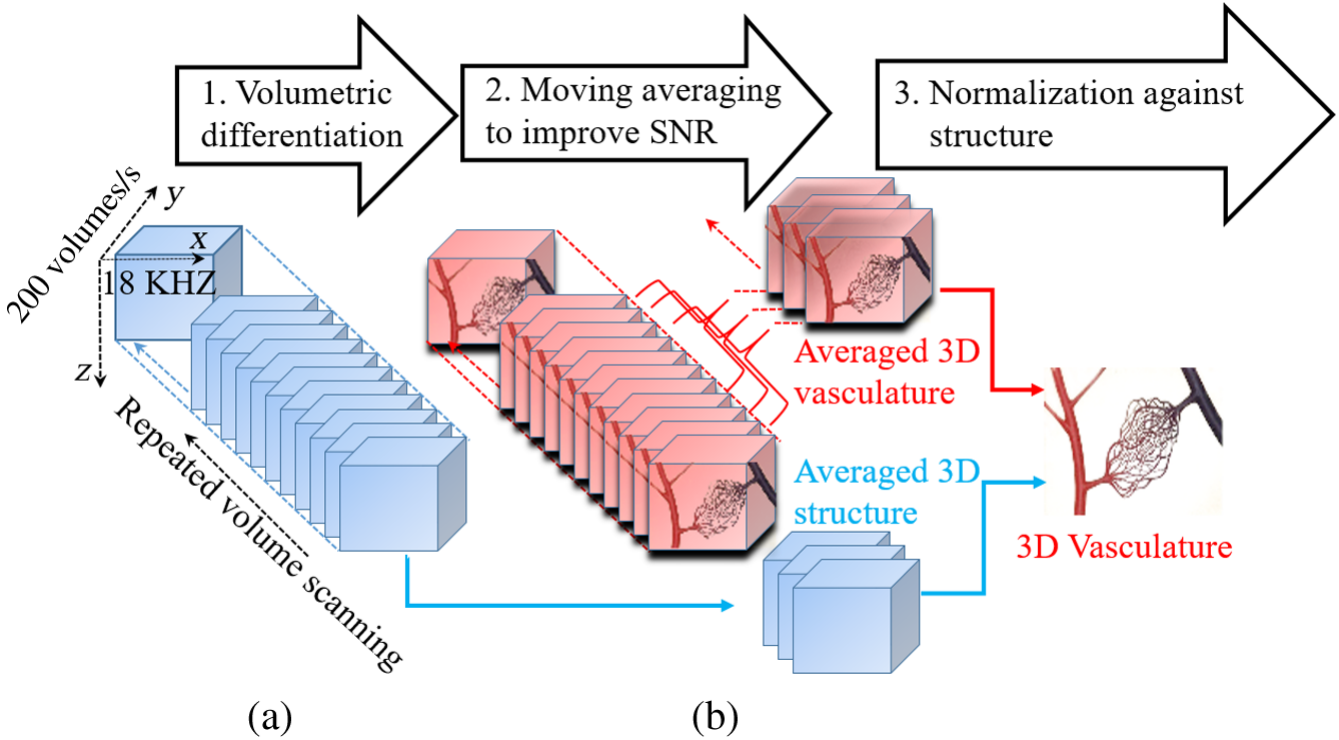
(a) vOMAG scanning protocol and (b) vOMAG angiography algorithm.

In order to achieve sensitive imaging of functional microvasculature from OCT structural datasets, the vOMAG algorithm is proposed, in which continuous differential operation is applied to the adjacent volumes with a time interval of ∼5  ms across all the volumes, giving a temporal resolution of ∼5  ms for 4-D angiography. To improve the signal-to-noise ratio (SNR), adjacent 3-D angiograms can be averaged depending on the temporal resolution required for different applications. In this demonstration, we used a moving average on every eight angiograms as an exemplar protocol in the following experiments. Correspondingly, adjacent nine 3-D structure datasets are also averaged for noise reduction.^9^ The vOMAG algorithm can be expressed as in Eqs. (1)–(2) and as illustrated in Fig. 2(b):

$$\overset{¯}{I_{\text{structure}}}=\frac{1}{N}\overset{N}{\underset{i=1}{\sum }}I_{\mathrm{Str}}(x,y,z,i),$$ (1)

$$\overset{¯}{I_{\text{Flow}}}=[1-\text{Norm}(\overset{¯}{I_{\text{Strucutre}}})]\frac{1}{N-1}\overset{N-1}{\underset{i=1}{\sum }}|I_{\mathrm{Str}}(x,y,z,i+1)-I_{\mathrm{Str}}(x,y,z,i)|,$$ (2)

where $I_{\mathrm{Str}}$, $\overset{¯}{I_{\text{Structure}}}$ and $\overset{¯}{I_{\text{Flow}}}$ are the intensities of the structure image, the averaged 3-D structure, and the averaged 3-D vasculature, respectively. (x,y,z) are the pixel coordinates corresponding to fast, slow, and depth scanning directions, respectively. N represents the number of volumes for averaging. Intensity-based analysis is used here because it is less sensitive to electronic trigger jitter, and thus is well suitable for SS-OCT.^14^ The OMAG image is normalized against the structure to remove hyperreflection artifacts, as calculated in the normalization operation $\left[1-\text{Norm}(\overset{¯}{I_{\text{Structure}}})\right]$ in Eq. (2). After that, 2-D cross-correlation is applied to the resulting OMAG images to further separate the bidirectional fast scans and to realign them together. A sinusoid resampling method is utilized to linearize the uneven sampling due to sinusoidal movement of the MEMS. Finally, a 4-D angiogram is reconstructed from the 3-D datasets for the direct observation of dynamic blood flow.

For high-speed OCT angiography, the temporal sensitivity is one of the key parameters to the detectable flow speed v, which is determined by the time interval Δt, according to the equation v=λ/2nΔt.^8^ Based on the scanning protocol above, the volume interval of ∼5  ms provides much higher temporal sensitivity than if performed on inter B-frame analysis where the time interval is approximately ∼0.056  ms. We assessed the sensitivity of vOMAG as compared to that of the interframe UHS-OMAG, in which the exact same postprocessing steps were applied to arrive at the final results. The designed experiment was similar to that in Ref. 8 for Brownian motion (BM) detection. A capillary tube with an inner diameter of ∼500  μm was submerged into a well-solidified background tissue phantom made of 8% gelatin and 0.02% ${\mathrm{TiO}}_{2}$. Ten percent of intralipid was sealed in the tube, which was positioned perpendicularly to the incident light. In the UHS-OMAG protocol, we performed nine repetitions of B-scans at each transverse location. The scanning protocol for vOMAG was the same as described above. The field of view (FOV) was $1\times 2\;{\mathrm{mm}}^{2}$ for both UHS-OMAG and vOMAG, which took ∼45  ms to finish the data acquisition. The imaging results are shown in Fig. 3, where Fig. 3(a) gives representative 3-D structure and cross-sectional structure of the fluid phantom. The corresponding vOMAG flow images and UHS-OMAG flow images are shown in Figs. 3(b) and 3(c), respectively, to indicate the BM of the particles. The blue circles and spline fitted lines in Fig. 3(d) shows comparison of the vOMAG signal (up) and UHS-OMAG signal (down) across the center of the tube [in the white dash line of Figs. 3(a)–3(c)]. It is clear that the sensitivity of vOMAG is much higher than that of the interframe UHS-OMAG under the current experimental setup.

**Fig. 3:**
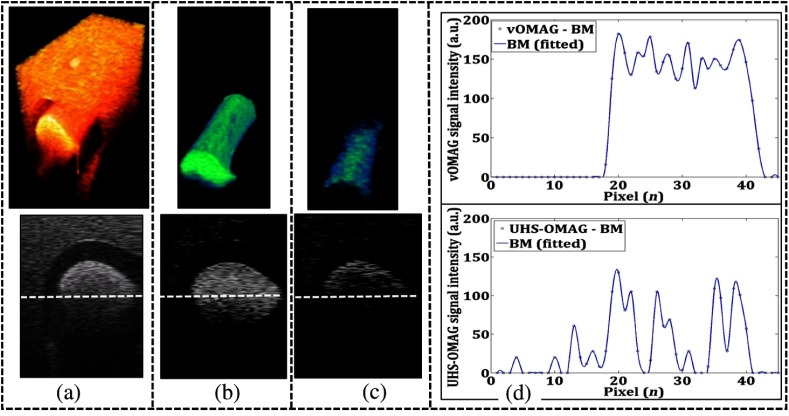
Assessment of vOMAG sensitivity to the BM as compared to interframe UHS-OMAG: (a) 3-D structure and cross-sectional structure images of a fluid phantom in which the intralipid scatters are not flowing. (b) Corresponding vOMAG flow images and (c) interframe UHS-OMAG flow images indicate the BM of particles. (d) Comparison of (up) the vOMAG signal and (down) the UHS-OMAG.

The vOMAG gives an SNR of 58 as compared to the SNR of 7.8 for the UHS-OMAG. The SNR improvement in vOMAG is attributed to its high sensitivity to flow due to Δt=∼5  ms versus ∼0.056  ms for UHS-OMAG to contrast the flow signal by data analysis. Another advantage for the vOMAG scanning protocol is that the slow scan axis can be furnished by a continuous ramp waveform, which provides a smooth scanning without the stabilization issues of step scanning as in the scanning protocol for conventional OCT angiography.

Averaging is an effective way to increase the SNR of resulting OCT angiograms. The SNR improvement in conventional OCTA is typically satisfied by the increase of B-scan repetition numbers at each transverse location, which, however, translates to the increased data acquisition time for each 3-D image. Such a coupled relationship between the total 3-D imaging time and the number of B-scan repetitions is indeed the current bottleneck for any conventional OCTA scanning strategies. However, such a coupling issue is relaxed for the vOMAG scanning strategy because there is no need to perform repeated B-scans at each transverse location in order to contrast the blood flow, but rather the analysis is performed on adjacent volumes. Therefore, given continuously acquired 4-D datasets over a certain time period, this decoupling feature in vOMAG offers at least two advantages: (1) the time interval Δt between volumetric datasets for analysis can be arbitrarily selected for different sensitivities of the system and (2) the volumetric averages can be conducted at as many volumes as possible for a high-definition and high-contrast 3-D angiography. Of course, such welcoming flexibility in vOMAG would depend on the 3-D temporal resolution required for specific investigations of vascular dynamics and its response to localized tissue injuries, for instance, in neurological diseases, such as stroke, Alzheimer’s disease, aging, and so on.^10^

To demonstrate the capability of vOMAG for high-quality 3-D angiography and high-speed 4-D angiography, we imaged the microcirculatory tissue bed in mouse brain *in vivo*. During imaging, a pulse oximeter (MouseOx plus, STARR) was synchronized to monitor mouse heartbeat through the left femoral artery. The scanning protocol described above was applied to continuously capture 100 volume datasets of mouse cerebral cortex through a cranial window within a 0.5-s time period. A high-definition 3-D vasculature is achieved by a moving average of the resulting 3-D angiograms. By mean intensity projection onto the x−y plane, the *en-face* oriented vascular networks are mapped as shown in Fig. 4(a). A cross-sectional structure image and corresponding blood flow image are shown in Figs. 4(b) and 4(c), respectively, where the blood vessels in the white matter can be clearly identified (yellow arrows). Within the same cross section, the vOMAG intensity profile across the small vessel [red dot in (a)] is plotted to show the spatial resolution of the vascular image, as shown in Fig. 4(d). Due to high temporal sensitivity and high vasculature contrast, blood vessels down to ∼30  μm (full width at half maximum) are recognizable, close to the limitation of the lateral resolution of the system.

**Fig. 4:**
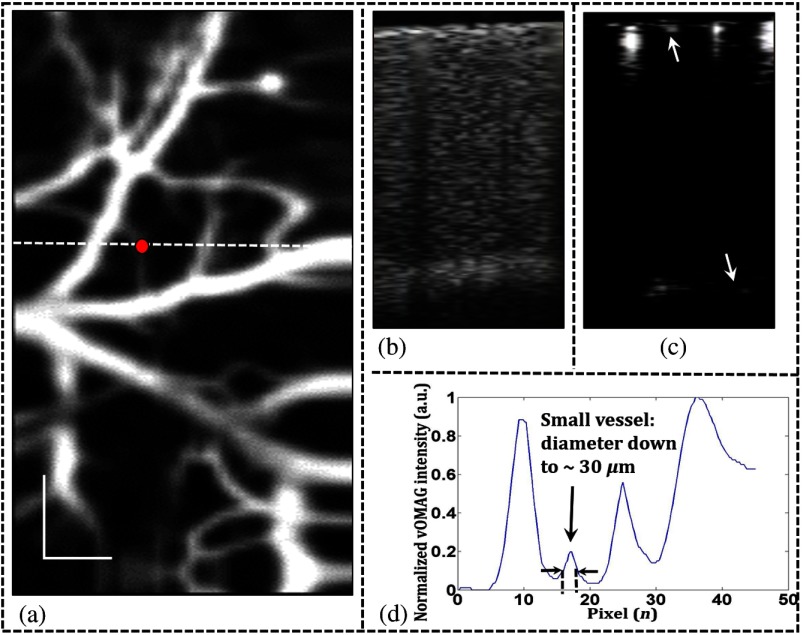
(a) *En-face* view of a 3-D vasculature (image FOV: $1\times 2\;{\mathrm{mm}}^{2}$) of mouse cerebral cortex. The dash line indicates the position of the cross-sectional images: (b) structure image and (c) blood flow image, as well as (d) a vOMAG intensity profile across a small vessel down to ∼30  μm [marked by dot in (a)]. In (c), the white arrow indicates the small vessel, and the yellow arrows indicate the blood vessels in the white matter. Scale bar: 200  μm.

To better show the flow dynamics, *en-face* views of the 4-D brain vasculature are streamed as a movie (visualization 1). Flow dynamics in the functional vessels as imaged by the system can be observed with high temporal resolution of ∼5  ms. Noting that the heartbeat rate of anesthetized mice is ∼8  Hz,^15^ the related vasculature change should be monitored at more than ∼16  Hz (Nyquist sampling). Giving the settings of the proposed system that gave 200  volumes/s, a moving averaging of maximal 12 adjacent volumes can be performed to improve the SNR of resulting images, while still sufficient to capture the dynamic blood flow due to heartbeat. In the demonstration to improve the temporal resolution, we instead implemented the moving averaging across eight continuous volumes for the 4-D angiography to reduce the effect of the heartbeat on the results. Frames were extracted from the resulting 4-D sequence to show flow dynamics of selected vessels (i.e., an arteriole, a venule, and one of the smallest vessels) as marked in Figs. 5(a) and 5(b). The vOMAG signal variations of these functional vessels are calculated and plotted along the time [Fig. 5(c)] to show the continuous dynamics when the blood cells are pushing through the vessels. The pulsatile flow dynamics in the arteriole^1^ and its small branch^3^ follow the mouse heartbeat, giving a repeated fluctuation of approximately 4 periods in 0.5 s. In contrast, there is no obvious pulsatile profile for the selected venule.^2^ The mouse heartbeat waveform from the oximeter (0.72-s period) is shown in the last subgraph of Fig. 5(d), in which the heartbeat rate is measured to be 436 bpm (∼7  beats/s). The measurement of heartbeat from the resulting vOMAG signal profile in the arteriole gave about 8  beats/s, which agreed well with the measurement from the pulse oximeter (Video 1).

**Fig. 5:**
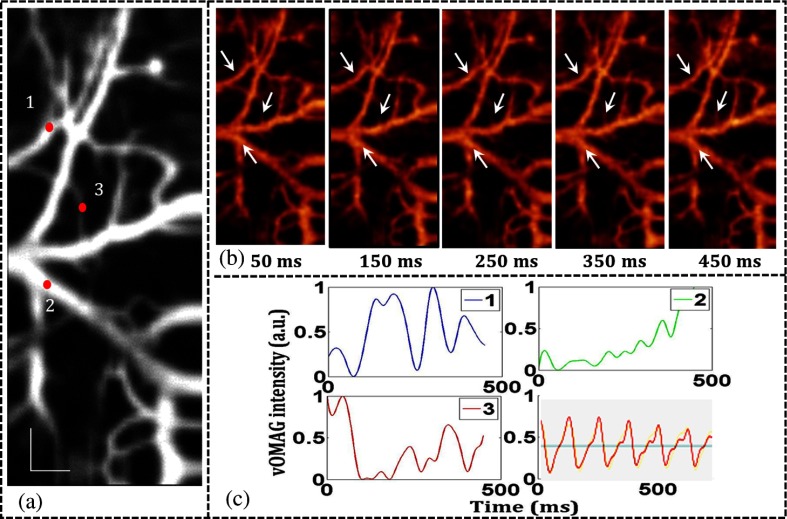
*En-face* view with sequential numbers indicating (1) an arteriole, (2) a venule, and (3) one of the smallest vessels, respectively. (b) Frames taken from the projected 4-D movie (visualization 1) to show time-varying blood flow dynamics. (c) vOMAG signal variations (spline fitted) to show blood flow dynamics in the functional vessels that marked in (b) and sequentially numbered in (a). Scale bar: 200  μm (Video 1, WMV, 1014 KB) [URL: http://dx.doi.org/10.1117/1.JBO.21.3.036005.1].

Another highlighted feature in our system is the highly integrated dual axial MEMS, which has been increasingly utilized for endoscopic OCT imaging.^16^ In the current demonstration, we only employed the fast scan axis to fulfill the purpose to achieve high-speed vOMAG angiography. Because of the limited size of the MEMS mirror ($1\times 1\;{\mathrm{mm}}^{2}$), the usable probe beam size was restricted, affecting the effective lateral resolution that can be achieved (∼29  μm). In the future, we plan to mitigate this problem through proper mechanical and optical designs in the sample arm so that the lateral resolution is improved for capillary imaging. In addition, the scanning FOV was confined within $1\times 2\;{\mathrm{mm}}^{2}$ with spacing of 22  μm between adjacent A-scans in the x−y directions. This is largely because the 1.62 MHz A-line rate is not able to support the requirement of more A-line samples for a wider FOV. Luckily, rapid development of swept laser sources sweeping at several tens of MHz^17^^,^^18^ has been recently reported. We expect that the future development of wide FOV vOMAG would be feasible if this new swept laser source is employed in the system. Finally, real-time OCT display has also been realized through a graphic processing unit to give video rate 3-D structures.^19^ Thus, vOMAG-based real-time 4-D angiography (including endoscopic implementation) can be expected to investigate ultrafast vascular dynamics within human body. Such a development would make the scenario become a reality where neuronsurgeons monitor 3-D brain microvasculature in real time on their computer screens while conducting delicate and precision brain surgery to patients.

In summary, we proposed a vOMAG method based on an intervolume analysis to contrast microvascular blood flow within scanned tissue volume, achieved by a high-speed OCT system featuring 1.62 MHz FDML SS and an effective 36 kHz MEMS scanner. We have demonstrated that the system is capable of 4-D OCT angiography at ∼200  volumes/s. In addition, the vOMAG scanning protocol and algorithm provide a practical and flexible way to achieve both high temporal sensitivity and high temporal resolution. The performance of vOMAG for high-definition 4-D microangiography has been demonstrated by monitoring the dynamic blood flow in mouse brain *in vivo*. For the first time, imaging speed, temporal sensitivity, and temporal resolution become sufficient for a direct 4-D observation of dynamic blood flow in small vessels.
